# Clinical Efficacy and Safety of Minimally Invasive Sacroiliac Joint Fusion: A Retrospective Cohort Study

**DOI:** 10.7759/cureus.110489

**Published:** 2026-06-08

**Authors:** Koleas Zumbro, Magdalena Falasca, Lawrence Maccree

**Affiliations:** 1 Neurosurgery, Lincoln Memorial University DeBusk College of Osteopathic Medicine, Harrogate, USA; 2 Neurosurgery, Methodist Medical Center, Oak Ridge, USA

**Keywords:** chronic low back pain, clinical outcomes, minimally invasive sacroiliac joint fusion, minimally invasive surgery, patient-reported outcome measures (proms), sacroiliac joint fusion, sacroiliitis, visual analog scale (vas)

## Abstract

Background

Sacroiliitis, inflammation of one or both sacroiliac (SI) joints, is a frequent yet often underrecognized source of chronic low back pain. Conservative treatments - such as physical therapy, medications, and corticosteroid injections - are the first-line treatment options for these patients. However, when they fail to provide lasting relief, surgery may become necessary. The purpose of this study is to evaluate the clinical outcomes of minimally invasive SI joint fusion (MI SIJF), specifically focusing on pain improvement and postoperative complication rates at the six-month follow-up.

Methodology

A retrospective chart review of 17 patients who underwent MI SIJF procedures was conducted. Two of the participants underwent bilateral SI fusions at separate time points, resulting in a total of 19 MI SIJF procedures in the cohort. Patient-reported outcomes, including pain scores via the visual analog scale (VAS), were collected at baseline, 10 days, six weeks, 10 weeks, and six months postoperatively. Complication rates and time to symptom relief were also recorded. Statistical analyses were performed using paired t-tests and linear mixed-effects modeling to assess changes in VAS pain scores across postoperative timepoints. A p-value of <0.05 was considered statistically significant. Analyses were performed using Microsoft Excel (Microsoft® Corp., Redmond, WA) and jamovi (Jonathon Love, Damian Dropmann, and Ravi Selker, Sydney, Australia).

Results

From the data collected, the average percent pain relief at six months post-SI fusion was 74.7%. The mean VAS pain score improved significantly from a baseline of 75.8 to 17.8 at six months. While initial symptom relief was noted as early as 10 days postoperatively, maximal improvement was realized by the six-month mark. No major complications were observed within the cohort.

Conclusion

This study suggests that MI SIJF is a safe and clinically effective treatment for patients with chronic sacroiliitis who have not achieved adequate relief through conservative management. The observation of significant pain reduction as early as 10 days postoperatively highlights a potential for early recovery that warrants further investigation. While further prospective controlled trials are necessary to validate these findings in larger populations, our results indicate that MI SIJF is a reliable intervention for achieving substantial symptomatic improvement in patients with chronic sacroiliitis.

## Introduction

The sacroiliac (SI) joint, which connects the sacrum and ilium of the pelvis, is a frequent yet often underrecognized source of chronic low back pain. In fact, it is estimated to account for 15% to 30% of non-radicular back pain cases [1,2]. Sacroiliitis, defined as inflammation of one or both SI joints, may arise from degenerative osteoarthritis, inflammatory spondyloarthropathies, trauma, or pregnancy-induced ligament laxity. When conservative treatments - such as physical therapy, medications, and corticosteroid injections - do not yield sufficient relief, surgery may be indicated.

SI joint fusion (arthrodesis) is the definitive surgical intervention for sacroiliitis, eliminating micro-motion by stabilizing and fusing the joint. Historically performed via an open approach with extensive soft-tissue dissection, the standard now favors minimally invasive SI joint fusion (MI SIJF) [3,4]. Typically, a lateral or posterior transiliac approach is used, placing titanium implants across the joint space. These implants provide immediate mechanical stability, with long-term fusion achieved through joint decortication and bone graft application.

Clinical evidence increasingly supports the efficacy of MI SIJF over continued conservative care. High-level evidence from the INSITE trial [5], a randomized controlled trial, demonstrated that surgical patients experienced a four-fold greater improvement in pain scores compared to those receiving non-surgical management. Furthermore, the iMIA study [6] demonstrates that approximately 78.8% of surgical patients had a reduction in visual analog scale (VAS) pain by 20 points or more at six months. Longitudinal data from Sachs et al. suggest that the success rate may be even higher, with 91.9% of patients achieving a “Substantial Clinical Benefit” (SCB), defined by the authors as either a reduction in VAS pain scores of more than 25 points or reaching a low absolute pain score of 35 or less at their 12-month follow-up [7].

The magnitude of pain reduction is equally compelling. Recent meta-analyses and response studies indicate an average pain reduction of 53.5% to 65.9% [5,6,8]. Notably, while some patients experience initial symptom relief as early as the six-week follow-up, maximal pain improvement and functional recovery are typically realized around six months postoperatively [9]. Supporting this, prospective data from the SIFI study [10] shows that 76% of patients achieve a significant improvement in disability - defined as a reduction in the Oswestry Disability Index (ODI) [11] of more than 10 points - by the six-month mark.

Despite these positive outcomes, the procedure is not without risk. Although minimally invasive surgical techniques have significantly reduced morbidity, complication rates still range from 1.0% to 9.2% [12], with the most common issues being transient nerve irritation, hematomas, or wound infections. With the increasing prevalence of SI joint fusion, it is critical to further investigate the nuances of postoperative recovery and the specific variables that predict long-term clinical success.

While clinical evidence supports the efficacy of MI SIJF, many of these studies utilize large, multi-center datasets that may not reflect the outcomes achieved in a specialized, single-center clinical practice. The primary aim of this retrospective chart review is to quantify pain reduction, as measured by VAS scores, following MI SIJF. Secondary aims include evaluating postoperative complication rates, characterizing the timeline to initial symptom relief, and determining the proportion of patients achieving both the Minimal Clinically Important Difference (MCID) and SCB over a six-month follow-up period.

## Materials and methods

Study design and ethics

This study was a retrospective chart review of 17 patients who underwent MI SIJF between January 2024 and August 2025. Two of the participants underwent bilateral SI fusions at separate time points, resulting in a total of 19 MI SIJF procedures in the cohort. The study was conducted in accordance with the Declaration of Helsinki and received Institutional Review Board exemption from the Covenant Health Institutional Review Board. A waiver of informed consent was granted due to the study's retrospective design and the use of de-identified patient data.

Patient selection criteria

Patients were identified via an electronic medical record (EMR) search using International Classification of Diseases, Tenth Revision (ICD-10) and Current Procedural Terminology (CPT) codes associated with sacroiliitis and SI joint fusion, along with the provider’s information. All of the patients in our cohort had never had an SI joint fusion on the same side prior to the one in question. Thus, none of the surgeries included in our cohort were SI fusion revisions. 

Inclusion and Exclusion Criteria

The study cohort comprised adult patients (≥18 years) presenting with chronic SI joint pain persisting for a minimum of six months. Patient selection was governed by a rigorous diagnostic and therapeutic algorithm aligned with current International Society for the Advancement of Spine Surgery (ISASS) policy recommendations [13].

Eligibility was strictly defined by the failure of comprehensive non-operative management. All patients were required to have completed a total course of conservative treatment spanning a minimum of three to six months prior to surgical consideration. This treatment plan included, at minimum, six weeks of intensive, supervised non-operative therapy - such as physical therapy, osteopathic manipulative treatment (OMT), or chiropractic care - in combination with optimized pharmacologic management (nonsteroidal anti-inflammatory drugs (NSAIDs)). In all cases, surgery was reserved for patients who failed to achieve significant functional improvement despite this multidisciplinary approach.

A confirmed diagnosis was required via physical examination, defined by at least three positive provocative tests, such as SI compression, anterior superior iliac spine (ASIS) distraction, Gaenslen’s, FABER (flexion, abduction, external rotation), thigh thrust, or Yeoman’s tests as established by clinical benchmarks [14,15]. In addition, both hip and lumbar pathologies were ruled out for all of our patients, as documented in the initial History and Physicals. 

Furthermore, all included patients needed to demonstrate a positive response to at least one fluoroscopically guided SI joint corticosteroid injection, characterized by a minimum of 70% pain reduction for at least two weeks. While this study focuses on the clinical outcomes of our initial cohort, a more comprehensive analysis of our patient selection protocol and the predictive value of these specific diagnostic criteria is the subject of a forthcoming manuscript.

Given our small sample size and the fact that we were only including SI fusions performed by one provider, we did not exclude any patients from our cohort. Therefore, all patients who underwent an MI SIJF between January 2024 and August 2025 by our provider were included in our cohort. Reasons for exclusion for surgical intervention would have been if they presented with an active systemic infection, a localized infection at the intended surgical site, or significant sacral or iliac bone tumors or fractures.

Surgical technique

All procedures were performed by a single surgeon using a lateral percutaneous minimally invasive approach. Patients were positioned in either the prone or lateral decubitus position under general anesthesia. Under fluoroscopic guidance utilizing lateral, inlet, and outlet views, lateral transiliac access was obtained. In each case, three hydroxyapatite-coated, self-drilling, and self-harvesting titanium screws (8.7 mm diameter; lengths ranging from 35 mm to 50 mm) were utilized for stabilization. The implants were advanced along a trajectory perpendicular to the SI joint. Criteria for optimal implant placement included careful visualization of the neural foramina, assessment of the anteroposterior (AP) diameter of the sacrum, and ensuring all hardware remained strictly below the alar line. Beyond the passage of the self-harvesting screws, which facilitated the collection of local autograft, no specific joint decortication was performed, and no additional allograft was used.

Postoperative protocol and perioperative management

Following the procedure, a standardized postoperative protocol was implemented for all patients. To minimize the risk of surgical site infection, patients were prescribed a 10-day course of oral cephalexin 500 mg to be taken three times daily (TID). Postoperative pain management was standardized with a three-day course of hydrocodone/acetaminophen (10 mg/325 mg) for acute analgesic control. Regarding mobility, patients were restricted to toe-touch weight-bearing (TTWB) for the first two postoperative weeks, during which the use of assistive devices - such as a walker or crutches - was permitted to maintain stability while ensuring no significant load was placed on the operative limb. After the initial two-week period, patients began a gradual weaning process from assistive devices, transitioning toward weight-bearing as tolerated (WBAT) based on clinical progression.

Data extraction and outcome measures

Clinical data were extracted from preoperative (before surgery) records and subsequent postoperative follow-up visits conducted at 10 days, six weeks, 10 weeks, and six months. The demographic variables extracted during our chart review included age, gender, body mass index (BMI), diabetes status, smoking status, and osteoporosis status. The primary clinical outcome measured was pain intensity, which was quantified using the VAS ranging from 0 (no pain) to 100 (worst pain imaginable). In addition, radiographic assessment of the SI fusion status, including implant position, loosening, migration, or malposition, was also evaluated using radiographs at follow-up visits.

In accordance with the iMIA/Dengler criteria [6], clinical success was defined as a reduction of 20 points or greater in the VAS score relative to the preoperative baseline. Additionally, the study monitored for procedure-related complications, encompassing all intraoperative and postoperative adverse events.

Statistical analysis

Statistical analyses were performed after data collection using paired t-tests to assess improvements in VAS scores following treatment. Paired t-tests were used to compare VAS scores at postoperative timepoints (10-day, six-week, 10-week, and six-month follow-up) with baseline scores. Given the small sample size, nonparametric Wilcoxon signed-rank tests were also performed as a sensitivity analysis. Effect sizes were calculated using Cohen’s d to quantify the magnitude of clinical change independent of sample size. Normality of paired differences was assessed using skewness, kurtosis, and Q-Q plots, which demonstrated approximate normal distributions at each time point. Additionally, the percent pain reduction and the proportion of patients achieving ≥50% pain improvement were evaluated at each time point.

Due to incomplete follow-up across timepoints, repeated-measures ANOVA was not performed; however, a linear mixed-effects model was used to account for repeated measures and missing data. To compare with previously published literature, one-sample t-tests were performed comparing individual six-month percent pain reduction values to established benchmark values (53.5%, 59.7%, and 65.9%). A p-value of <0.05 was considered statistically significant. All analyses were performed using Microsoft Excel (Microsoft® Corp., Redmond, WA) and jamovi statistical software (Jonathon Love, Damian Dropmann, and Ravi Selker, Sydney, Australia).

## Results

Patient demographics and baseline VAS scores

A total of 19 MI SIJFs were performed across 17 patients. Table 1 provides the demographic characteristics of all the patients in our cohort. For the two patients who underwent staged bilateral procedures, each surgery was treated as an independent observation for the purpose of clinical and statistical analysis. However, patient-level clustering was accounted for using a random intercept for patients in the linear mixed-effects model. All patients included in the analysis had a confirmed diagnosis of SI joint dysfunction, underwent MI SIJF, and completed 10-day, six-week, 10-week, and six-month follow-up visits.

**Table 1:** Demographic Characteristics of the Study Population *Calculated based on 19 independent surgical observations.

Characteristic	Value (N = 19*)
Age (years)	
Mean (± SD)	65.5 ± 10.1
Median (Range)	65 (47-82)
Gender, n (%)	
Male	10 (52.6%)
Female	9 (47.4%)
Diabetes Status, n (%)	
Diabetics	10 (52.6%)
Non-diabetics	9 (47.4%)
BMI, n (%)	
Underweight (<18.5)	0 (0.0%)
Healthy Weight (18.5-24.9)	0 (0.0%)
Overweight (25.0-29.9)	6 (31.6%)
Obese (≥30.0)	13 (68.4%)
Osteoporosis Status, n (%)	
Diagnosed With Osteoporosis	1 (5.3%)
No Known Osteoporosis	18 (94.7%)
Smoking Status, n (%)	
Current or Former Smoker	13 (68.4%)
No Smoking History	6 (31.6%)

The mean initial VAS pain score in this group at time zero was 75.8 ± 12.7 (95% CI, 69.2-81.4). Table 2 breaks down the specific VAS score reported by each patient in our cohort at their initial History and Physical (baseline VAS) as well as their reported VAS score at their 10-day, six-week, 10-week, and six-month follow-up visits. For the two patients who had bilateral SI fusion surgeries, they were treated as separate time points, given the fact that they had independent History and Physical exams and follow-up visits for each of their SI fusion surgeries.

**Table 2:** Breakdown of Clinical Outcomes for Each Patient Included in Our Cohort Note: Patient numbers with asterisks (*) represent the two patients who underwent bilateral staged SI fusions during our study period. Dashes (-) represent follow-up visits that the patient did not attend.

Patient Number	Baseline VAS	10-Day VAS	6-Week VAS	10-Week VAS	6-Month VAS
1	80	60	80	50	30
2	70	20	60	40	40
3	40	30	40	30	20
4	80	50	-	0	0
5	85	30	10	10	10
6	70	20	20	30	30
7	50	50	30	20	10
8	80	-	30	20	30
9*	95	0	0	0	0
10*	90	30	0	-	0
11	80	-	30	20	20
12*	80	10	-	0	0
13*	80	0	0	0	0
14	80	50	20	10	10
15	80	50	50	40	40
16	80	60	40	20	20
17	70	40	70	70	60
18	80	60	-	30	0
19	70	40	40	20	-

Outcomes

A retrospective chart review was conducted on 17 patients (representing 19 discrete surgical procedures) who underwent MI SIJF. While all 17 patients attended postoperative follow-up, the total number of clinical observations at each interval varied. Reasons for incomplete follow-up included both missed appointments and patients lost to follow-up.

A paired analysis of our six-month follow-up showed that the mean VAS pain score improved from 75.8 ± 12.7 (95% CI, 69.2-81.4) preoperatively to 17.8 ± 17.7 (95% CI, 9.0-26.6). This represents a statistically significant 74.7% reduction in pain (p < 0.05). The paired effect size was large (Cohen’s d = 2.36; 95% CI, 1.86-2.86), indicating a substantial pain reduction following SI joint fusion. In addition, 17 (94.4%) patients achieved the MCID (which is defined as a greater than or equal to 20-point improvement in VAS score) at the six-month follow-up.

Pain reduction and success rates

Postoperative records show a mean pain (VAS) reduction of 74.7% at six months for the cohort. Nonparametric analysis using Wilcoxon signed-rank testing confirmed significant reductions in VAS scores at each time point, including 10 days (p = 0.0005), six weeks (p = 0.0016), 10 weeks (p = 0.0003), and six months (p = 0.0002). A linear mixed-effects model demonstrated a significant effect of time on VAS scores (F = 34.0, p < 0.001). Compared to baseline, VAS scores were significantly reduced at all postoperative timepoints, including 10 days (β = -41.2, p < 0.001), six weeks (β = -44.9, p < 0.001), 10 weeks (β = -54.5, p < 0.001), and six months (β = -58.8, p < 0.001). The model demonstrated strong explanatory power (marginal R² = 0.563; conditional R² = 0.712), with an intraclass correlation coefficient of 0.342, indicating moderate between-patient variability.

Using the definition described by the iMIA study [6] of an MCID (greater than or equal to 20-point reduction in VAS pain), this chart review identified a 94.4% success rate at six months. We also calculated the number of patients who received an SCB described by Sachs et al. [7], which is quantified by either a reduction in VAS pain of more than 25 points or reaching a low absolute pain score of 35 or less. Using this definition, our chart review found that 17 (94.4%) patients received an SCB from their SI fusion surgery at six months. 

Clinical timeline

Review of postoperative follow-up data and documentation revealed a notably rapid onset of pain relief. Our review found that patients consistently reported initial symptomatic improvement as early as 10 days postoperatively, as seen with a mean pain reduction of 50.6%, with seven (41.2%) patients reporting at least 50% improvement at that time. This early clinical benefit was sustained throughout the six-month review period.

Table 3 provides the mean VAS scores, calculated MCID, and mean pain reduction for the 10-day, six-week, 10-week, and six-month follow-up visits. The number of patients who attended these follow-ups is reported as n, and the p-values for each mean VAS score are also reported in Table 3. Most significantly at six months, mean pain improved from 75.8 ± 12.7 (95% CI, 69.2-81.4) preoperatively to 17.8 ± 17.7 (95% CI, 9.0-26.6). This corresponded to a mean pain reduction of 74.7% ± 25.1%, with 14 (77.8%) patients at that time period achieving ≥50% pain improvement.

**Table 3:** Observed Clinical Outcomes Following Sacroiliac Joint Fusion Note: Mean pain reduction % at each time point was calculated by taking the average ((preoperative pain - postoperative pain)/(preoperative pain))*100. MCID is defined as a reduction in VAS pain greater than or equal to 20 relative to the preoperative baseline. Percentages are calculated based on the total number of clinical observations (n) available at each specific follow-up interval. The p-values are in reference to the results of paired t-tests performed comparing the VAS score of each postoperative visit to baseline. Abbreviations: MCID, Minimal Clinically Important Difference; n, number of observations; Preop, preoperative; VAS, visual analog scale

Metric	Preoperative Baseline	10 Days	6 Weeks	10 Weeks	6 Months
Follow-Up (n)	-	17	16	18	18
Mean VAS Score	75.8 ±12.7	35.3 ± 20.0	32.5 ±24.4	22.8 ±19.0	17.8 ±17.7
Mean Pain Reduction (%)	-	50.6 ±28.9	52.8 ± 25.9	67.1 ± 28.3	74.7 ± 25.1
MCID, n (%)	-	15 (88.2%)	12 (75.0%)	16 (88.9%)	17 (94.4%)
p-value	-	p < 0.05*	p < 0.05*	p < 0.05*	p < 0.05*

Safety and complications

The review of surgical and follow-up records showed an overall complication rate of 1 (5.3%) in our cohort. This lone complication was classified as superficial dehiscence, which is the partial separation of a previously sutured surgical incision edge. This responded to a prophylactic 10-day course of cephalexin 500 mg TID and resolved completely without any additional management. No instances of infection, nerve irritation, hematomas, implant mispositioning/migration, or surgery revisions were identified within the six-month follow-up window.

The average operation time for our cohort was 40.1 ± 5.6 minutes (mean ± SD), and there was a “minimal” amount of blood loss reported for each surgery as well. A “minimal” amount of blood loss was defined as either not requiring suction or insignificant. 

Case illustration

To demonstrate the clinical application of our diagnostic algorithm and subsequent surgical outcomes, we present the case of a 58-year-old male with an eight-month history of debilitating left-sided SI joint pain (preoperative VAS 85). At the initial presentation, the patient exhibited functional limitations, including impaired ambulation, intolerance to prolonged sitting or standing, and significant difficulty with stair climbing, lifting, carrying, and rising from a forward-flexed position.

Physical examination revealed a positive Fortin finger test over the left SI joint. Provocative maneuvers were highly demonstrative, with positive findings on thigh thrust, FABER, Gaenslen’s, and Yeoman's tests. Secondary lumbar spine and hip pathologies were systematically evaluated and ruled out via focused physical examination. Advanced imaging demonstrated radiographic evidence of spondyloarthropathy without alternative structural etiologies for the patient's pain.

In accordance with our treatment protocol, the patient completed four months of total conservative management. This included a six-week course of structured physical therapy, which yielded no benefit, alongside optimized pharmacologic management. Subsequently, the patient underwent a fluoroscopically guided left SI joint corticosteroid injection, which provided him with greater than 70% pain relief for greater than two weeks.

**Figure 1:**
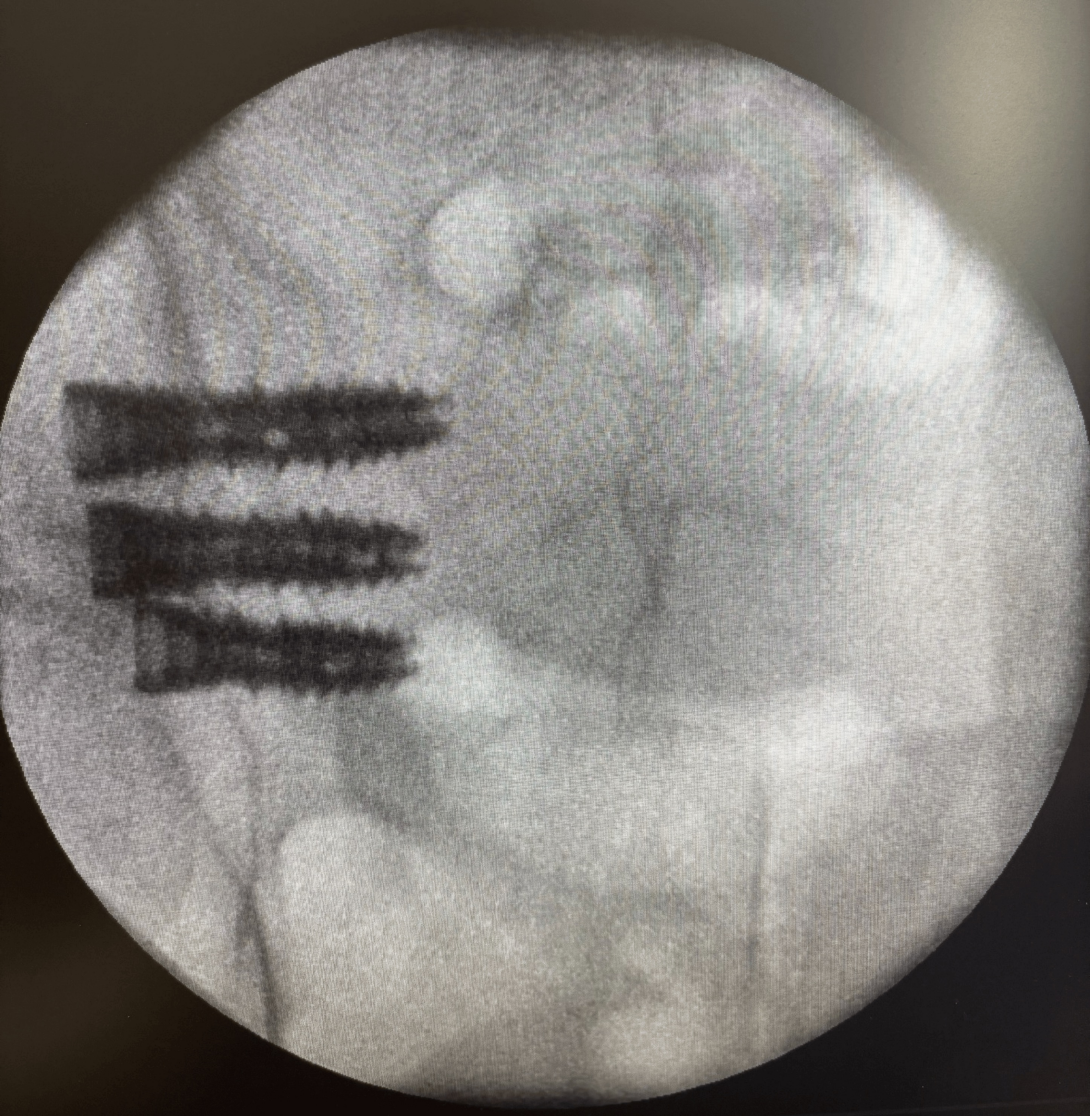
Intraoperative Fluoroscopic Pelvic Outlet View Fluoroscopic visualization of the implants confirming their position relative to the sacral foramina, ensuring no neural encroachment.

**Figure 2:**
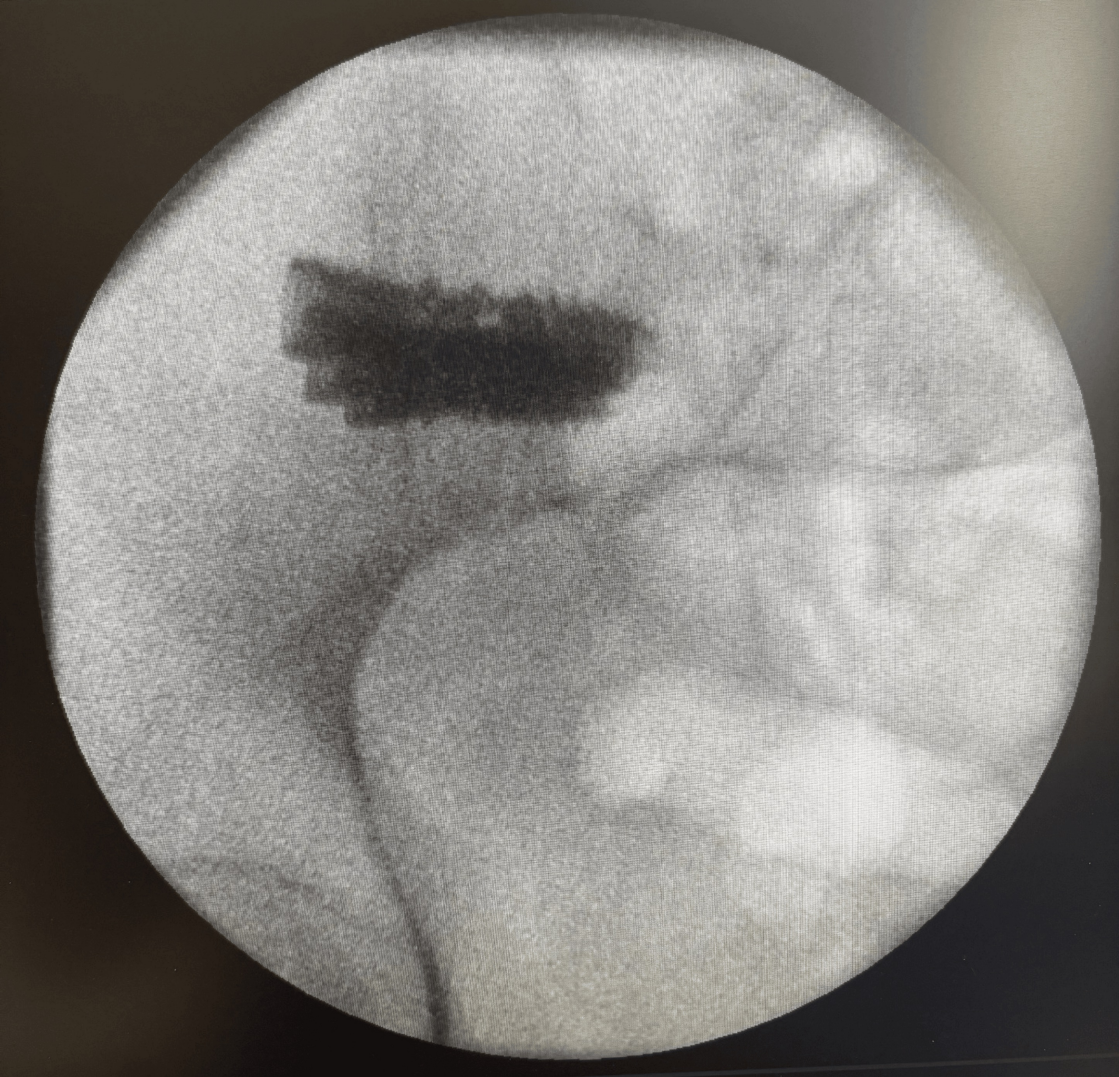
Intraoperative Fluoroscopic Pelvic Inlet View Fluoroscopic imaging demonstrating the trajectory of the three triangular titanium implants across the sacroiliac joint.

**Figure 3:**
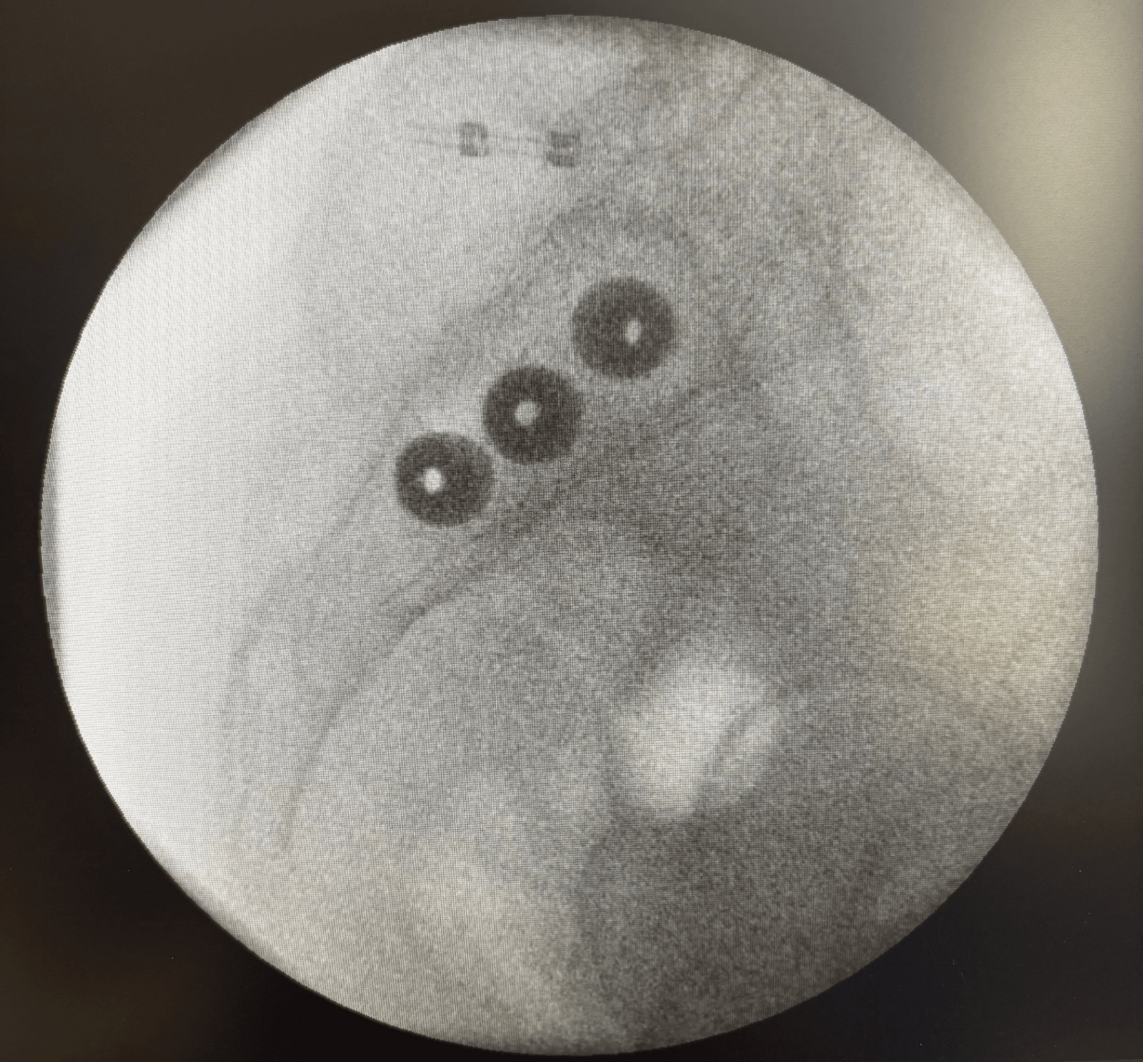
Intraoperative Fluoroscopic Lateral View Lateral fluoroscopic view confirming that the implants are contained within the sacral bone, avoiding breach of the anterior or posterior sacral cortex.

Following the failure of this four-month conservative regimen, the patient underwent MI SIJF utilizing three triangular titanium implants. Precise intraoperative trajectory and stable implant placement across the joint line were confirmed using intraoperative fluoroscopic outlet, inlet, and lateral views (Figures 1-3).

**Figure 4:**
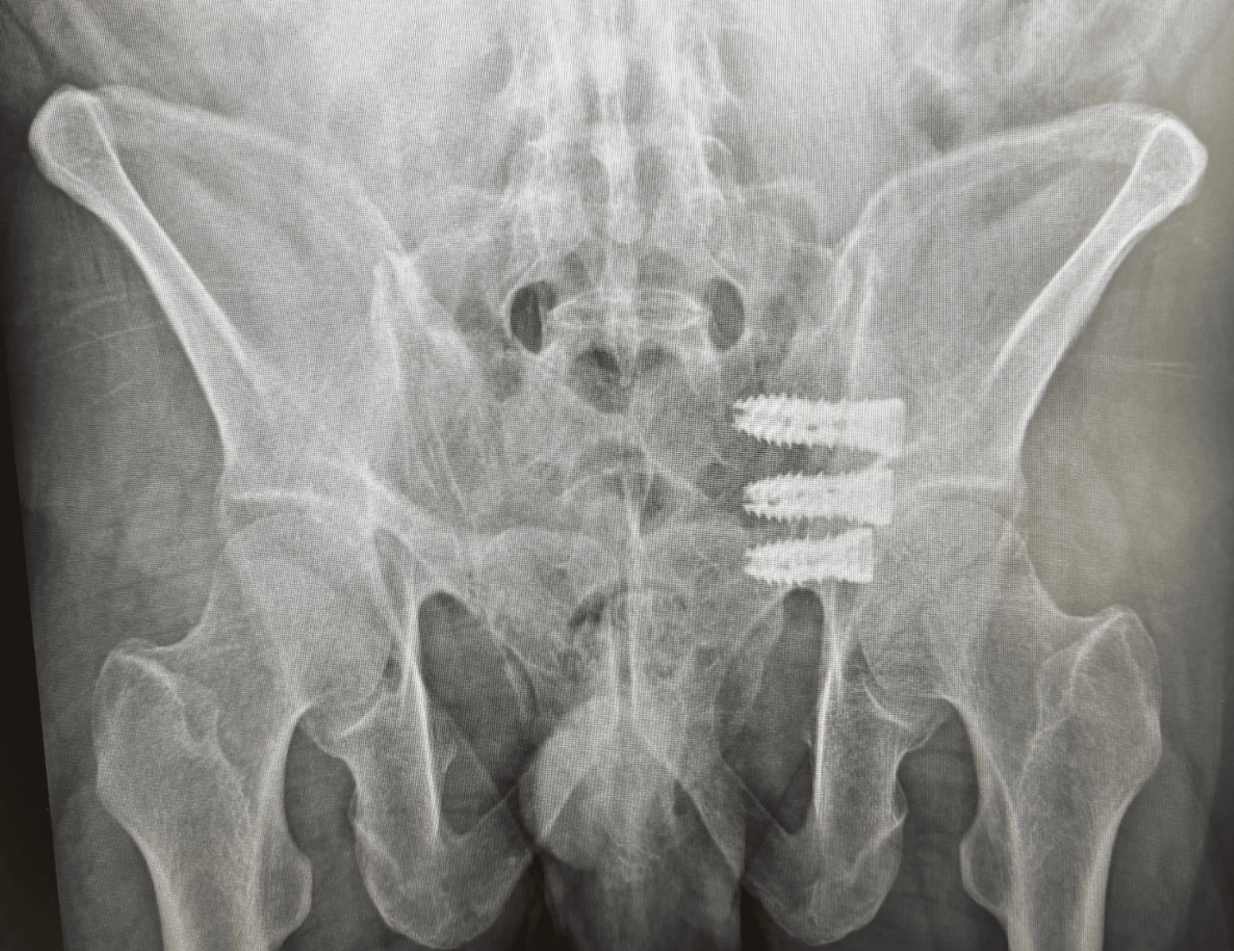
Postoperative Pelvic Outlet Radiograph Follow-up imaging at six months showing maintained implant position. The implants remain stable with no evidence of migration or lucency.

**Figure 5:**
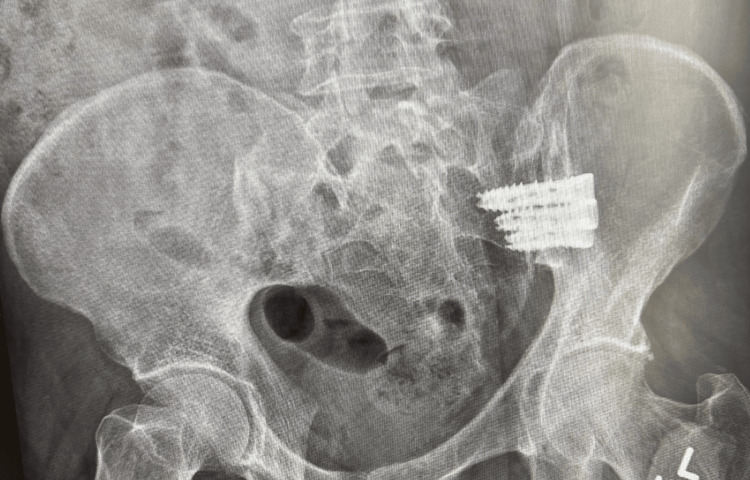
Postoperative Pelvic Inlet Radiograph Six-month follow-up imaging demonstrating maintained structural integrity of the implants and successful alignment within the ilium and sacrum.

**Figure 6:**
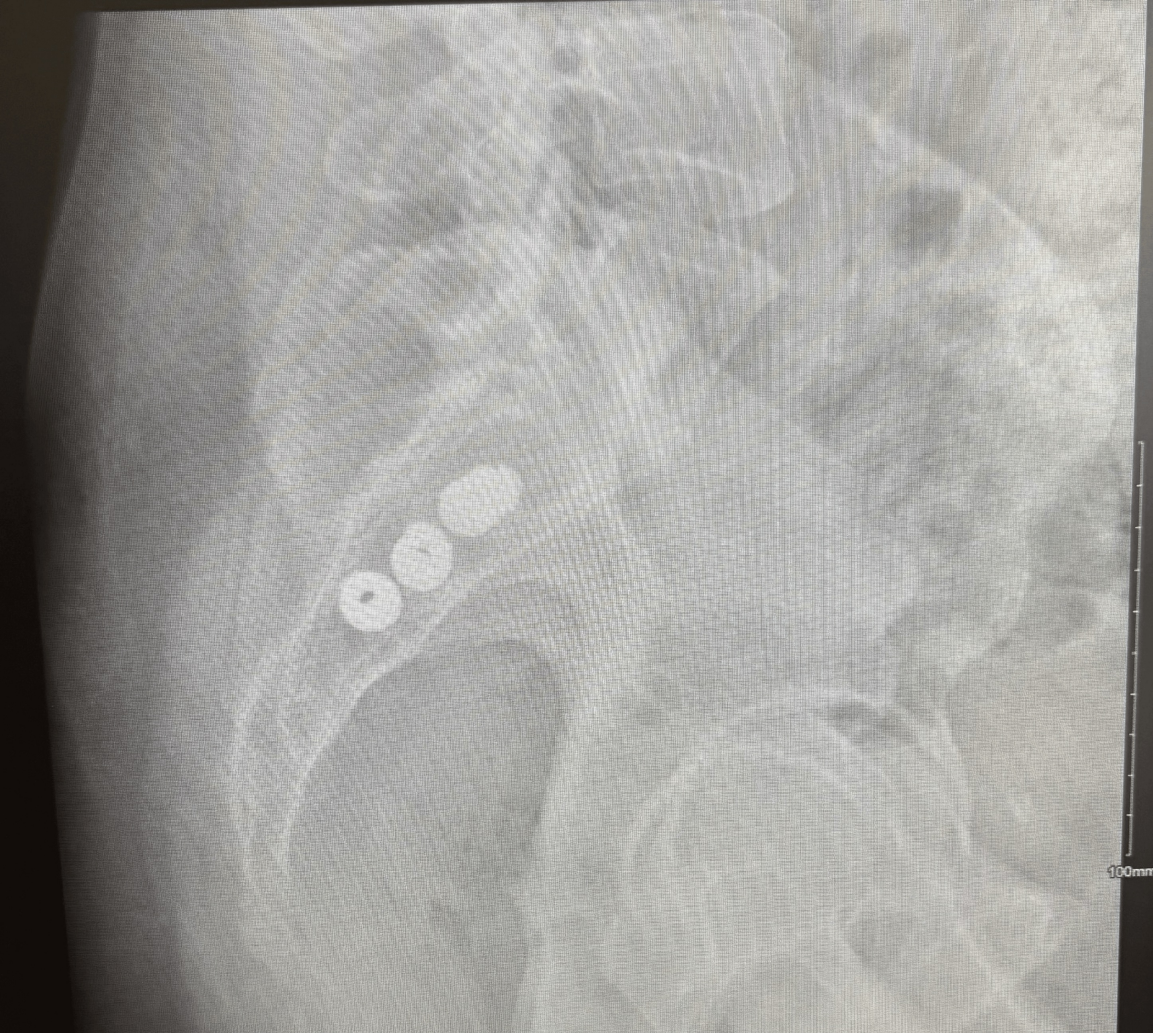
Postoperative Lateral Radiograph Postoperative lateral view showing stable implant positioning at six months postoperatively. No signs of hardware loosening, subsidence, or migration are observed.

At the 10-day postoperative follow-up, the patient’s pain scores decreased substantially to a VAS of 30, and he successfully discontinued all opioid analgesics. At the six-month follow-up interval, the patient reported high clinical satisfaction, a VAS score of 10, and a complete return to independent activities of daily living. Radiographic imaging at six months (Figures 4-6) demonstrated excellent hardware stability and no evidence of peri-implant lucency, implant loosening, or hardware migration.

## Discussion

The clinical outcomes of this study demonstrate that MI SIJF leads to significant and sustained pain reduction in patients with confirmed SI joint dysfunction. Our cohort achieved a mean VAS pain reduction of 74.7% at six months, a result that compares favorably to - and in some metrics exceeds - benchmarks established in current literature (Table 4).

**Table 4:** Comparison of Observed Clinical Outcomes to Literature Benchmarks Note: MCID is defined as a reduction in VAS pain greater than or equal to 20 relative to the preoperative baseline. SCB is defined as either a reduction in VAS pain of more than 25 points or reaching a low absolute pain score of 35 or less. Percentages are calculated based on the total number of clinical observations (n) available at each specific follow-up interval. Abbreviations: MCID, Minimal Clinically Important Difference; SCB, Substantial Clinical Benefit; VAS, visual analog scale

Metric	Our Study Results	Literature Benchmark	Reference
Success Rate (MCID)	94.4%	78.8%-90.0%	iMIA study and Sachs et al. [6,7]
Success Rate (SCB)	94.4%	91.9%	Sachs et al. [7]
Mean Pain Reduction (VAS)	74.7%	53.5%-65.9%	ISITE trial, iMIA study, Calodney et al. [5,6,8]
Significant Relief (Time)	10 days-6 months	6 weeks-6 months	EvoluSIon study [9]

Pain reduction, success rates, and clinical significance

This retrospective chart review of patients undergoing MI SIJF demonstrates clinical outcomes that consistently exceed established multicenter benchmarks. The most notable finding is the 74.7% mean reduction in VAS pain scores observed at the six-month follow-up. This outcome surpasses the benchmark range of 53.5%-65.9% established by the INSITE trial [5], the iMIA study [6], and recent meta-analytic data [8]. One-sample t-testing demonstrated that this improvement was significantly greater than both the lower bound (53.5%) and midpoint (59.7%) (p < 0.05), but did not reach statistical significance when compared to the upper bound (65.9%), likely due to variability within the cohort and the small sample size. The 95% confidence interval for mean pain reduction overlapped with the upper bound of reported values, further explaining the lack of statistical significance in that comparison.

Using the definition of an MCID (greater than or equal to 20-point reduction in VAS pain), this chart review identified a 94.4% success rate at six months. This clinical outcome, detailed in Table 4, surpasses the 78.8% MCID reported in the iMIA randomized controlled trial [6] and the 90.0% MCID observed by Sachs et al. [7].

We also calculated the number of patients who received an SCB described by Sachs et al. [7], which is quantified by either a reduction in VAS pain of more than 25 points or reaching a low absolute pain score of 35 or less. Using this definition, our chart review found that 17 (94.4%) patients received an SCB from their SI fusion surgery at six months. This clinical outcome, detailed in Table 4, surpasses the 91.9% SCB observed by Sachs et al. [7].

These results suggest that the surgical technique and patient selection criteria employed in this series provide a highly predictable and significant clinical benefit. The specific diagnostic workup and preoperative selection criteria utilized for this cohort will be the subject of a forthcoming investigation.

Comparison with landmark trials

Our findings align with the results of the INSITE and iMIA randomized controlled trials, which reported surgical success as being superior to non-surgical management [5,6]. While these trials reported a mean VAS reduction of approximately 53.5% to 65.9% at six months, our cohort demonstrated a more pronounced reduction of 74.7%. This discrepancy may be attributed to our stringent preoperative selection process, which required at least 70% pain reduction for a minimum of two weeks to at least one diagnostic SI joint corticosteroid injection - a higher threshold than the 50% pain relief from transient anesthetic blocks used for inclusion in the INSITE and iMIA studies [5,6].

Recent evidence suggests that short-acting anesthetic blocks like those used in the randomized controlled trials may be susceptible to false positives due to the placebo effect or anesthetic leak into the lumbosacral plexus [16]. Unlike the snapshot provided by local anesthetics, a response to corticosteroids requires a biological modulation of joint inflammation. Schneider et al. [17] suggest that a sustained response to anti-inflammatory modulation provides a more stable indicator of the joint as a chronic pain generator. By more precisely isolating the SI joint as the primary pain generator, we likely improved the predictive value of the surgical intervention.

Accelerated recovery timeline

A distinguishing feature of this study is the rapid onset of symptom relief. While the EVoluSIon study [9] identifies a recovery curve typically beginning between six weeks and six months, patients in our cohort reported significant relief as early as 10 days postoperatively.

This accelerated timeline is a critical finding, as it suggests that the immediate mechanical stability provided by the transiliac implants may offer clinical benefits much sooner than previously emphasized in prospective trials. While maximal recovery is often associated with the maturation of biological fusion around the six-month mark [9], our data indicate that the primary breakthrough in pain relief can occur within the first two weeks of the postoperative period, as seen with a mean pain reduction of 50.6% at 10 days postop, with seven (41.2%) patients reporting at least 50% improvement at that time. While this finding could be due to the restrictions placed on patients for the first 10 days postoperatively, we believe it still suggests a clinical benefit before the six-week timeline established by the EVoluSIon study [9].

Radiographic and hardware stability

Radiographic evaluation was performed for all 17 patients (19 procedures) during the postoperative follow-up period using SI joint radiographs. Direct visualization of the hardware confirmed proper initial implant positioning in all cases. At the final radiographic follow-up, there were no observed instances of implant loosening, migration, or malpositioning. Furthermore, no evidence of periprosthetic lucency or hardware failure was identified within the cohort. While the timing of follow-up imaging varied, the available radiographic data consistently demonstrated mechanical stability of the SI joint fusions throughout the study duration.

Safety and complications

The review of surgical and follow-up records showed an overall complication rate of 5.3% in our cohort. This falls within the expected safety profile of 1.0% to 9.2% reported by the systematic review by Xu et al. [12]. This lone complication was classified as superficial dehiscence, which is the partial separation of a previously sutured surgical incision edge. This responded to a prophylactic 10-day course of antibiotics and resolved completely without any additional management.

Although the INSITE trial [5] found a 1.5-2.5% rate of infection from this procedure, there were no instances of infection, either superficial or deep, observed in our cohort. Additionally, there were no documented cases of nerve irritation or hematomas observed in our cohort, while Xu et al. [12] reported these complications in their systematic review. Finally, there was no documentation of implant mispositioning or migration, nor were there any instances of surgery revisions identified within our six-month follow-up window. These complications were mentioned in both Sachs et al. and Xu et al. [7,12].

Limitations

While the clinical outcomes of this study are compelling, several limitations must be acknowledged to provide a balanced interpretation of the data. First, the retrospective nature of this chart review introduces potential selection and information bias. Second, the sample size (n = 19) is small compared to large multicenter trials, which may limit the generalizability of the results. Third, the six-month follow-up period, while consistent with the window for biological fusion maturation, does not capture the long-term durability of the implants beyond the first six months. Fourth, two patients underwent bilateral staged procedures, which may introduce a correlation between observations. This was addressed statistically using patient-level random intercepts in the mixed-effects model, although residual bias remains possible given the small sample size. Finally, because all procedures were performed by a single surgeon, these results may reflect a high level of technical consistency that might vary across different surgical settings.

Additionally, the absence of a control group or a non-operative comparator arm represents a significant limitation, as it prevents the formulation of definitive comparative efficacy claims regarding the superiority of MI SIJF over conservative management. Furthermore, this study focused primarily on pain reduction as measured by the VAS; the absence of objective functional outcomes or quality-of-life metrics, such as the ODI, represents a limitation that should be addressed in future prospective research.

## Conclusions

This study suggests that MI SIJF is a safe and clinically effective treatment for patients with chronic sacroiliitis who have not achieved adequate relief through conservative management. The observation of significant pain reduction as early as 10 days postoperatively highlights a potential for early recovery that warrants further investigation. While further prospective controlled trials are necessary to validate these findings in larger populations, our results indicate that MI SIJF is a reliable intervention for achieving substantial symptomatic improvement in patients with chronic sacroiliitis.
